# Cellular expansion of MSCs: Shifting the regenerative potential

**DOI:** 10.1111/acel.13759

**Published:** 2022-12-19

**Authors:** Katherine Miclau, William S. Hambright, Johnny Huard, Martin J. Stoddart, Chelsea S. Bahney

**Affiliations:** ^1^ Center for Regenerative and Personalized Medicine (CRPM) Steadman Philippon Research Institute Vail Colorado USA; ^2^ Orthopaedic Trauma Institute (OTI) University of California San Francisco San Francisco California USA; ^3^ AO Research Institute Davos Davos Switzerland

**Keywords:** cellular senescence, in vitro expansion, mesenchymal stromal cell, MSC, passaging, regeneration, secretome

## Abstract

Mesenchymal‐derived stromal or progenitor cells, commonly called “MSCs,” have attracted significant clinical interest for their remarkable abilities to promote tissue regeneration and reduce inflammation. Recent studies have shown that MSCs' therapeutic effects, originally attributed to the cells' direct differentiation capacity into the tissue of interest, are largely driven by the biomolecules the cells secrete, including cytokines, chemokines, growth factors, and extracellular vesicles containing miRNA. This secretome coordinates upregulation of endogenous repair and immunomodulation in the local microenvironment through crosstalk of MSCs with host tissue cells. Therapeutic applications for MSCs and their secretome‐derived products often involve in vitro monolayer expansion. However, consecutive passaging of MSCs significantly alters their therapeutic potential, inducing a broad shift from a pro‐regenerative to a pro‐inflammatory phenotype. A consistent by‐product of in vitro expansion of MSCs is the onset of replicative senescence, a state of cell arrest characterized by an increased release of proinflammatory cytokines and growth factors. However, little is known about changes in the secretome profile at different stages of in vitro expansion. Some culture conditions and bioprocessing techniques have shown promise in more effectively retaining the pro‐regenerative and anti‐inflammatory MSC phenotype throughout expansion. Understanding how in vitro expansion conditions influence the nature and function of MSCs, and their associated secretome, may provide key insights into the underlying mechanisms driving these alterations. Elucidating the dynamic and diverse changes in the MSC secretome at each stage of in vitro expansion is a critical next step in the development of standardized, safe, and effective MSC‐based therapies.

## Graphical abstract

Throughout in vitro expansion, MSCs and their secretome undergo a shift from a pro‐regenerative to a proinflammatory phenotype. Changes underlying this shift include alterations in DNA methylation profile, morphological abnormalities, telomere shortening, oxidative stress, senescence, and secreted biomolecules (cytokines, chemokines, growth factors, MSC‐EVs). Approaches that have shown promise in retaining the functional properties of culture‐expanded MSCs include cellular preconditioning (hypoxia, inflammatory cytokines, 3D culturing), pharmacological interventions (senolytics), and telomerase overexpression.

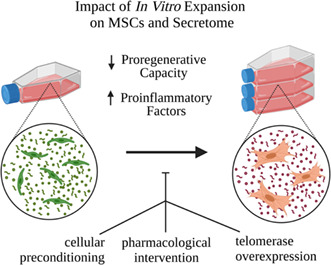

## 1 INTRODUCTION

Mesenchymal‐derived stromal cells (MSCs) have drawn significant interest in the past several decades for their therapeutic benefits in regenerative medicine, most notably due to their ability to reduce inflammation and repair tissue in injury and disease. MSCs are a heterogeneous population of unspecialized cells that can be isolated from several tissues within the body, including adipose tissue, bone marrow, dermal, skeletal muscle, umbilical cord, amniotic fluid, Wharton's jelly, placentae, and synovial fluid (Squillaro et al., 2016). As defined by the International Society for Cellular Therapy (ISCT), the minimal criteria to define mesenchymal stromal cells are: (1) plastic‐adherent in standard culture conditions; (2) a specific marker expression profile (CD105+, CD73+, CD90+, CD45–, CD34–, CD14– or CD11b–, CD79α– or CD19–, HLA‐DR–); and (3) potential for in vitro trilineage differentiation (adipocyte, chondrocyte, osteoblast; Dominici et al., 2006). In 2019, the ISCT MSC committee offered a subsequent position statement to clarify the nomenclature of mesenchymal stromal cells (MSCs) and include additional recommendations to consider tissue‐specific properties, rigorous evidence of in vitro and in vivo stemness, and a robust matrix of functional assays evaluating MSC properties when determining the applicability of the term MSC a cell population (Viswanathan et al., 2019). It should be highlighted, these were considered minimal criteria and their presence does not confirm a stem like progenitor cell.

Since the first MSC clinical trial in 1995, over 1500 human MSC‐based clinical trials have been registered on https://www.clinicaltrials.gov/ with wide‐ranging applications, including, multiple sclerosis, Crohn's disease, respiratory tract infections, myocardial infarction, graft‐vs‐host disease, and osteoarthritis (search term: “mesenchymal (stem OR stromal) cell”; “Home ‐ ClinicalTrials.Gov,” n.d.). MSCs are currently the most commonly used cells in regenerative medicine (González‐González et al., 2020). These MSC‐based therapies include both the autologous or allogenic intravenous administration of MSCs and several MSC‐derived biologics, such as MSC secretome and purified exosomes (Konala et al., 2016). At the time of writing, there are 10 globally approved MSC‐based products (Table 1; BioInformant Worldwide, LLC, n.d.; Childs et al., 2020).

**TABLE 1:** Ten globally approved MSC‐based therapies.

MSC therapy	Company	Clinical indication	Country (year) of approval	Cell type	Type	Dose size
Queencell	Anterogen Co. Ltd.	Subcutaneous tissue defect	South Korea (2010)	Human AT‐MSC	Autologous	7*10^7^ cells
Cellgram‐AMI	Pharmicell Co. Ltd.	Acute MI	South Korea (2011)	Human BM‐MSC	Autologous	50–90 m cells
Prochymal (Remestemcel‐L)	Osiris Therapeutics Inc./Mesoblast Ltd.	GvHD	Canada (2012) New Zealand (2012)	Human BM‐MSC	Allogenic	2 m cells/kg (10 doses)
Cupistem	Anterogen Co. Ltd	Crohn's Fistula	South Korea (2012)	Human AT‐MSC	Autologous	160 m cells
Cartistem	Medipost Co. Ltd.	Knee Cartilage Defects	South Korea (2012)	Human UC‐MSC	Allogenic	5 m cells/m
Neuronata‐R	Corestem Inc.	Amyotrophic lateral sclerosis	South Korea (2014)	Human BM‐MSC	Autologous	1 m cells/kg (every 2 weeks)
Temcell HS	JCR Pharmaceuticals	GvHD	Japan (2015)	Human BM‐MSC	Allogeneic	2 m cells/kg (12 doses)
Stempeucel	Stempeutics Research PVT	Critical limb ischemia	India (2016)	Human BM‐MSC	Allogeneic	2 m cells/kg
Alofisel	TiGenix NV/Takeda	Complex anal fistulas in CD	Europe (2018)	Human AT‐MSC	Allogenic	120 m cells
Stemirac	Nipro Corp.	Spinal cord injury	Japan (2018)	Human BM‐MSC	Autologous	50–200 m cells

Despite the success of achieving regulatory approval, many MSC‐based therapies have failed to show effectiveness in Phase III clinical trials or have not progressed to early pre‐clinical stages (García‐Bernal et al., 2021; Levy et al., 2020). Although MSC‐based therapies have shown promise for a wide variety of applications, significant variation exists in the outcomes of these treatments.

The ability of MSCs to influence tissue regeneration, remodeling, inflammation control, and cellular recruitment has also made them a primary target of tissue engineering and cell‐based treatments (Konala et al., 2016). MSC and MSC‐derived products have been shown to be safe with minimal immunogenicity upon injection (Squillaro et al., 2016), and MSC‐based therapies have exerted pro‐proliferative, anti‐inflammatory, pro‐angiogenic, and anti‐apoptotic functions (Boulestreau et al., 2020; Turinetto et al., 2016). The therapeutic efficacy of MSCs is currently believed to be attributed to several mechanisms, including MSCs' ability to home to sites of inflammation after tissue injury, to differentiate into various cell types, to secrete multiple bioactive molecules capable of stimulating recovery of injured cells and inhibiting inflammation and to perform immunomodulatory functions with low immunogenicity (Squillaro et al., 2016). However, MSCs' regenerative properties are compromised over time in in vitro culture, which has significant clinical implications (Baxter et al., 2004; Fehrer et al., 2006; Zhang et al., 2021). Furthermore, the current cell characterization methods do not reflect the increased attention on their secretome and immunomodulatory abilities, with a lack of functional predictive markers being a hurdle for their clinical translation.

### 1.1 Mechanism of action: Paracrine signaling as a driver of MSC therapeutic benefits

**FIGURE 1:**
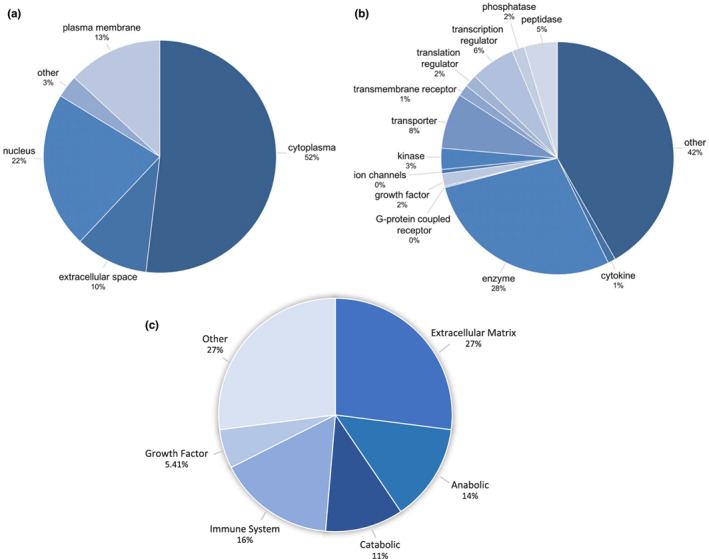
Protein composition of the MSC Secretome. The proteome of the MSC secretome is composed of proteins from diverse locations and cell functions. (a) Mass spectrometry analyses indicate that 52% located to the cytoplasm, 22% in the nucleus, 13% in the plasma membrane, and 10% in the extracellular space. (b) The breakdown of protein functionality is equally diverse: 28% enzymes, 8% transporter proteins, 6% transcription regulators, 5% peptidases, 3% kinases, 2% growth factors, and 2% translation regulators, with the largest fraction (42%) being responsible for highly diversified, unspecified functions (Kehl et al., 2019). (c) Proteomic analyses have revealed MSC secretome proteins group into functional categories of extra‐cellular matrix, anabolic, catabolic, immunoregulatory, growth factors, and other proteins (Wangler et al., 2021). Figure was created from combining data from Kehl et al. (2019; part a and b) and Wangler et al. (2021; part c).

While initially believed that upon implantation MSCs differentiate directly into specialized cell types to replace dead and damaged cells native to that tissue, growing evidence suggests that the observed therapeutic benefits in MSC therapies lie within the bioactivity of the collection of factors and molecules secreted by implanted MSCs (Phelps et al., 2018). Unlike in vitro culture, where MSCs can be induced to differentiate into a wide variety of cell types, the direct differentiation into specialized cell types at the site of the injury is less frequently observed in vivo. Findings support that frequently <1% of transplanted cells are retained long term within the target tissue in vivo, challenging the notion that the observed therapeutic benefits of MSC‐therapies are due to engraftment and differentiation (Haque et al., 2015; Tögel et al., 2005). Instead, implanted MSCs appear to accelerate healing and tissue repair by homing in on the sites of injury or disease and secreting a collection of molecules, including bioactive trophic and immunomodulatory factors, commonly referred to as the secretome (Caplan, 2017; Figure 1). Collectively, these paracrine factors appear to be responsible for the majority of MSCs' anti‐inflammatory and pro‐regenerative effects (Ahangar et al., 2020; Levy et al., 2020), with estimates as high as 80% of regenerative potential attributed to the secretome as opposed to the effect of direct differentiation (Maguire, 2013).

The term secretome encompasses two different components of the trophic factors released by MSCs: the soluble and the vesicular fraction. The soluble fraction includes cytokines, chemokines, immunomodulatory molecules and growth factors (Ferreira et al., 2018; Table 1). Of the soluble fraction, proteomic analyses have identified the presence of up to 1275 proteins involved in a variety of biological processes in BMSC conditioned media (Tachida et al., 2015). The broad array of these secreted factors act via different therapeutic mechanisms spanning anti‐inflammatory responses, anti‐apoptosis, mitogenesis, ECM formation, pro‐angiogenesis, anti‐fibrosis and enhancement of migration & homing (Table 2).

**TABLE 2:** Soluble factors associated with regenerative properties of MSCs

Anti‐inflammation	IDO, PGE2, TGFβ, IGF‐I, IL‐10, HGF, TSG6, nitric oxide, Hemo‐oxygenase, HLA‐G	García‐Bernal et al. (2021), Kouroupis et al. (2019)
Anti‐apoptosis	VEGF, HGF, IGF‐I, STC‐1, TGF‐β, GM‐CSF	da Silva Meirelles et al. (2009)
Mitogenesis	SCF, LIF, M‐CSF, SDF‐1, angiopoietin‐1	da Silva Meirelles et al. (2009)
ECM formation	MMPs, TIMP, ICAM, collagens, elastin, laminin	Ahangar et al. (2020)
Angiogenesis	VEGF, MCP‐1, FGF, Ang‐1, Ang‐2, angiostatin, PDGF, EGF, MMPs, GM‐CSF, CXCL5, IL‐8	Ahangar et al. (2020), Kim et al. (2018)
Anti‐fibrosis	HGF, PGE2, FGF	da Silva Meirelles et al. (2009), Dong et al. (2015)
Migration & Homing	PDGF‐AB, IGF‐1, RANTES, MDC, SDF‐1	Markov et al. (2021)

The vesicular fraction of the MSC secretome is composed of three major subtypes of extracellular vesicles (EVs) classified based on size and biogenesis: exosomes (40–150 nm in diameter), microvesicles (100–1000 nm), and apoptotic bodies (>1000 nm) (Almeria et al., 2022). EVs are phospholipid membrane‐bound particles whose cargo includes DNA, protein‐coding and non‐coding RNAs (mRNA, miRNA, lncRNA, circRNA), and small quantities of soluble bioactive molecules (Phelps et al., 2018). The lipid bilayer protects the EV content, directs transport through extracellular fluid, and facilitates internalization into recipient cells. Upon binding to appropriate receptors on target cells through receptor‐ligand interaction, EVs enter cells through the uptake pathways of signaling, fusion and endocytosis. EVs act both by releasing their cargo into the cytoplasm of target cells and by interacting with the membrane‐bound ligands with receptors expressed on target cells to influence downstream intracellular pathways (Maumus et al., 2020). Analyses of the contents of MSC‐derived EVs identified 730 different proteins and 171 miRNAs that regulate many specific pathways and biological processes; in total, mass spectrometry and antibody arrays show that purified MSC exosomes contain 938 unique gene products.

The use of MSC‐derived, cell‐free therapeutics presents several potential benefits relative to the injection of the cells themselves. Both MSC‐conditioned medium (CM) and purified MSC‐derived extracellular vesicles are able to influence local cell activity to promote endogenous repair via immunomodulation, anti‐inflammatory activity, angiogenesis, and anti‐apoptosis (Konala et al., 2016). This non‐soluble fraction plays an important role in cellular homeostasis and diffusion of biomolecules to neighboring cells and tissues and are considered to play particularly critical role in cell‐to‐cell communication (Teixeira & Salgado, 2020; Zhou et al., 2017). As of November 2022, there are 127 registered clinical trials on https://www.clinicaltrials.gov/ of EVs worldwide (search term: “extracellular vesicle”). Of these clinical trials, 42 are related to MSC‐derived exosomes or extracellular vesicles (search term: “mesenchymal (stem OR stromal) cells (exosomes OR extracellular vesicles)” (“Home ‐ ClinicalTrials.Gov,” n.d.)). Although many of these trials are in early phases and further research is required to determine their clinical effectiveness (only 2 registered as Phase 3 clinical trials and no phase 4 trials), the rapidly growing interest into cell‐free MSC‐based therapies reinforces the critical role the MSC secretome plays in healing and regeneration.

In addition to the regenerative and anti‐inflammatory effects, MSC‐based therapies have been shown to have an immunosuppressive function through interactions with both innate and adaptive immune systems. Early studies demonstrated MSC capacity to prevent the rejection of allogenic baboon skin grafts in vivo and inhibit T‐lymphocyte proliferation (Bartholomew et al., 2002). These immunosuppressive effects on T‐cell proliferation held true even when cell–cell contact between effector cells (T lymphocytes and dendritic cells) and BMSCs was inhibited, suggesting a critical role of the secretome in immunosuppression (Di Nicola et al., 2002). Further research elucidated how MSCs can affect a wide range of immunological responses through cell–cell contact, soluble factors, or exosomes. These include improving the lifespan, increasing phagocytosis capacity, and decreasing apoptosis of neutrophils (Taghavi‐Farahabadi et al., 2021). MSCs enhance the immune function of neutrophils' inflammatory factor expression modulated by the release of IL‐8, MIF, TSG6, IL‐10, and NO; these findings are reinforced through studies showing how MSCs derived from myeloma patients activate and transform neutrophils into an immunosuppressive and proangiogenic phenotype (Zheng et al., 2022). MSCs also inhibit the activation of proinflammatory M1 macrophages and promote the polarization of macrophages to the anti‐inflammatory M2 phenotype (Holthaus et al., 2022; Stevens et al., 2020), inhibit the proliferation, cytotoxicity, and cytokine production of natural killer cells (Spaggiari et al., 2008), suppress the proliferation, activation, and differentiation of T cells into pro‐inflammatory Th1 and Th17 helper cells while simultaneously increasing the activity of regulatory T lymphocytes (Luz‐Crawford et al., 2013), inhibit the differentiation and maturation of dendritic cells (Spaggiari et al., 2009), and suppress the proliferation and maturation of B cells (Wang, Wang, et al., 2018).

## 2 BIOPROCESSING CONSIDERATIONS FOR MSC‐BASED CLINICAL APPLICATIONS

Although MSC‐based therapies have shown promise for a wide variety of applications, significant variation exists in the outcomes of these treatments. A variety of MSC‐based therapies have been employed clinically, including both the autologous or allogenic intravenous administration of MSCs and several MSC‐derived biologics, including MSC secretome and purified exosomes (Parekkadan & Milwid, 2010). Despite the success of these therapeutics with regulatory approval, many MSC‐based therapies have failed to show effectiveness in late‐stage clinical trials or have not progressed beyond early pre‐clinical stages (Levy et al., 2020). Factors contributing to the heterogeneity of clinical outcomes of MSC‐based therapies include variations in preparation and manufacturing, route of administration, clinical target, trial design, assessment methodology, and the recipients of MSCs or their derivatives.

While the ideal number of infused cells in regenerative therapies remains unknown, most clinical therapies utilize a quantity of cells far greater than can be extracted straight from human tissue, where MSCs are present at a much lower frequency (Drela et al., 2019). For two of the most commonly sourced tissues, bone marrow, and adipose tissue, estimates of MSCs range from 0.001% to 0.01% of mononuclear cells (Bonab et al., 2006; Pittenger et al., 1999; Wexler et al., 2003) and 1% to 5% of nucleated cells (Zhu et al., 2008), respectively. Yet, protocols range anywhere from 10 to 400 million MSCs per treatment (Estrada et al., 2013; Haque et al., 2015). Achieving sufficient numbers of cells for these treatments often requires an in vitro expansion model of the isolated MSCs compared with an intraoperative model (Bara et al., 2014), which is a timely and expensive process. Techniques for this cellular expansion are dependent on dose and whether cells have autologous (requiring scale out manufacturing via personalized small batches) or allogenic (requiring scale‐up of one or a few large batches) applications. Furthermore, for cell‐free therapeutics, the number of MSCs required to produce sufficient quantities of secretome is estimated to be 10–25 times that of directly administering cells, resulting in even more extensive in vitro expansion (Ahangar et al., 2020).

However, standardizing bioprocessing considerations is complicated by the inherent heterogeneity of MSC cell populations and the lack of a single characteristic or marker, which poses challenges for a universal definition of MSCs (Keating, 2012). While the current panel of markers as outlined by the ISCT‐proposed minimal criteria is useful for basic MSC characterization, it is insufficient in delineating a reliable and robust cGMP batch release criteria for clinical purposes. As outlined in a review by Robb et al. (2019), “surface markers do not merely serve for the purpose of cell identification but have important biological roles in cell functions that we must understand to develop a functionally relevant panel of markers to characterize MSC quality and predict therapeutic performance of the cell product” (Robb et al., 2019). Alternative methods for batch release quality control, such as assessments of the cell transcriptome, proteome, and secretome, should also be considered in the future re‐evaluation of the MSC discrimination and functional potency (Ranganath et al., 2012). For example, commonly used batch release potency assays include the in vitro inhibition of T‐cell proliferation with activated CD4+ T cells to assess immunomodulatory bioactivity of MSC products (Levy et al., 2020). Furthermore, in addition to gaining a better understanding of MSC characteristics and functionality, standardization of nomenclature is necessary to increase consistency, and regulations of MSC research and clinical applications (Keating, 2012). Currently, MSCs have been referred to as Marrow Stromal Stem Cells (Owen, 1988), Medicinal Signaling Cells (Arnold I. Caplan, 2017), Mesenchymal Stem Cells (Caplan, 1991), and Mesenchymal Stromal Cells (Dominici et al., 2006).

Despite attempts to better define the MSC phenotype, many factors impact the therapeutic efficacy of cells through the process of in vivo expansion. The therapeutic potential of MSCs and their secretome is influenced by variations across donor (age, gender, disease, and metabolic state), tissue source (adipose and bone marrow), within‐donor sampling population, method of mononuclear cell (MNC) isolation, density media, centrifugation process, and the duration of cell attachment (Bara et al., 2014). Although age has been noted as one of the greatest sources of variation, significant, non‐age‐attributed differences exist between MSCs from different donors, resulting in up to 12‐fold differences between growth properties of BMSCs from healthy patients (Phinney et al., 1999). The culture expansion conditions also have a critical influence on the phenotype and function of MSCs, including culture medium (DMEM versus αMEM), glucose levels, media supplements (fetal bovine serum versus human platelet lysate versus serum‐free media), seeding density, oxygen level (normoxia, hypoxia, anoxia), platform (flasks, suspension bioreactors, hanging drop spheroids), preconditioning to pro‐inflammatory cytokines, temperature and exposure to electromagnetic, and biochemical and mechanical stimuli (Phelps et al., 2018; Yang et al., 2018). In addition to these discrepancies in origin and bioprocessing methods, the freezing, storage, and in vivo delivery methods (days, confluency, population doublings) differ across laboratories and protocols, rendering it even more challenging to compare findings across studies (Phelps et al., 2018). All these factors can alter the therapeutic potential of MSCs and introduce significant variation in the outcome of clinical trials.

With the increase in FDA clinical trials with a diverse array of bioprocessing protocols, efforts have been made to standardize MSC bioprocessing across clinical trials in order to ensure the production of safe, regulated, and efficient cell therapy products. Efforts to improve quality control measures and reporting standard resulted in the development of a task force to establish guiding principles for Good Cell Culture Practice (GCCP; Coecke et al., 2005; Hartung et al., 2002), which were subsequently revised in 2018 to consider key developments and maturations of new technologies used in in vitro cell culture (Pamies et al., 2018). Certain features of the cell substrate have been highlighted by the FDA as critical considerations in the research and development of manufactured biological products; these include proper documentation of the exact source of cells, source laboratory, tissue or organ of origin, age/gender/medical history of donor, method of isolation, in vitro culturing procedures and materials, number of population doublings, and quality assurance of the cells (Food and Drug Administration, HHS 1998). The FDA has implemented a set of regulations requiring compliance with current Good Manufacturing Practices (GMP) and Good Tissue Practices in the United States. Additionally, MSC therapies are considered advanced therapy medicinal products (ATMPs) by the European Medicines Agency by regulation No. [EC] 1394/2007 of the European Commission and must be produced in compliance with Good Manufacturing Practices.

While defined GMP standards are a means of ensuring sterility, quality control, and documentation (Fekete et al., 2012), there are significant differences in GMP‐compliant expansion protocols of FDA clinical trials. Of the MSC‐based product submissions as of 2015, about 90% used atmospheric oxygen, 80% used FBS, 25% used growth factors in addition to serum, and 80% use cryopreservation to store and transport the final product and 35% use cell banking systems (Mendicino et al., 2014). Clinical‐grade MSCs expanded in facilities with differences in GMP processes produced lots with significant variation in MSC characteristics (Menard et al., 2013). Principal component and unsupervised clustering analyses consistently separated lots from different facilities into distinct clusters (Liu et al., 2017). In fact, differences in expansion methodology between sites accounted for more transcriptome variation than donor source (Stroncek et al., 2020). Thus, compliance with various GMP protocols does not ensure uniformity in properties of culture‐expanded MSCs.

The ability of the MSC secretome to recapitulate many of the properties associated with MSCs themselves has opened new opportunities for the development of cell‐free therapeutics as an alternative to cell‐based treatments (Ferreira et al., 2018). Bioprocessing benefits of cell‐free therapeutics include the ability to scale to specific dosages, improved storage and transportation, lower immunogenicity, better biocompatibility, decreased cost of production, and increased potential to evaluate the safety, efficacy, and consistency of products similar to other pharmaceutical products. In particular, exosomes have recently been investigated as a non‐cellular regenerative construct with the potential to engineer specific biomodifications to enhance circulation time, lower rate of clearance and degradation, and better protect its cargo (Mentkowski et al., 2018; Murphy et al., 2019). The possibility to tailor the biological product for desired cell‐specific effects for each treatment is a particularly promising prospect.

However, there remain significant limitations in our understanding of the proteomic composition of the MSC secretome, the activity and half‐life of its biomolecules, and the content of extracellular vesicles (miRNA, proteins, and mRNA), which renders bioprocessing challenging. Among current studies, there exists no comprehensive list of biomolecules that compose the MSC secretome. Furthermore, the relative contribution of the different factors to the therapeutic response is unclear. Similarly, to cell‐based products, utilizing the MSC secretome currently often requires in vitro expansion of MSCs to produce enough conditioned medium for therapeutic use, a process that may significantly alter both the MSC phenotype and its affiliated secretome profile (Phelps et al., 2018).

## 3 FUNCTIONAL AND PHENOTYPIC CHANGES IN MSCS THROUGHOUT IN VITRO EXPANSION

### 3.1 Overview of changes throughout in vitro expansion of MSCs

**FIGURE 2:**
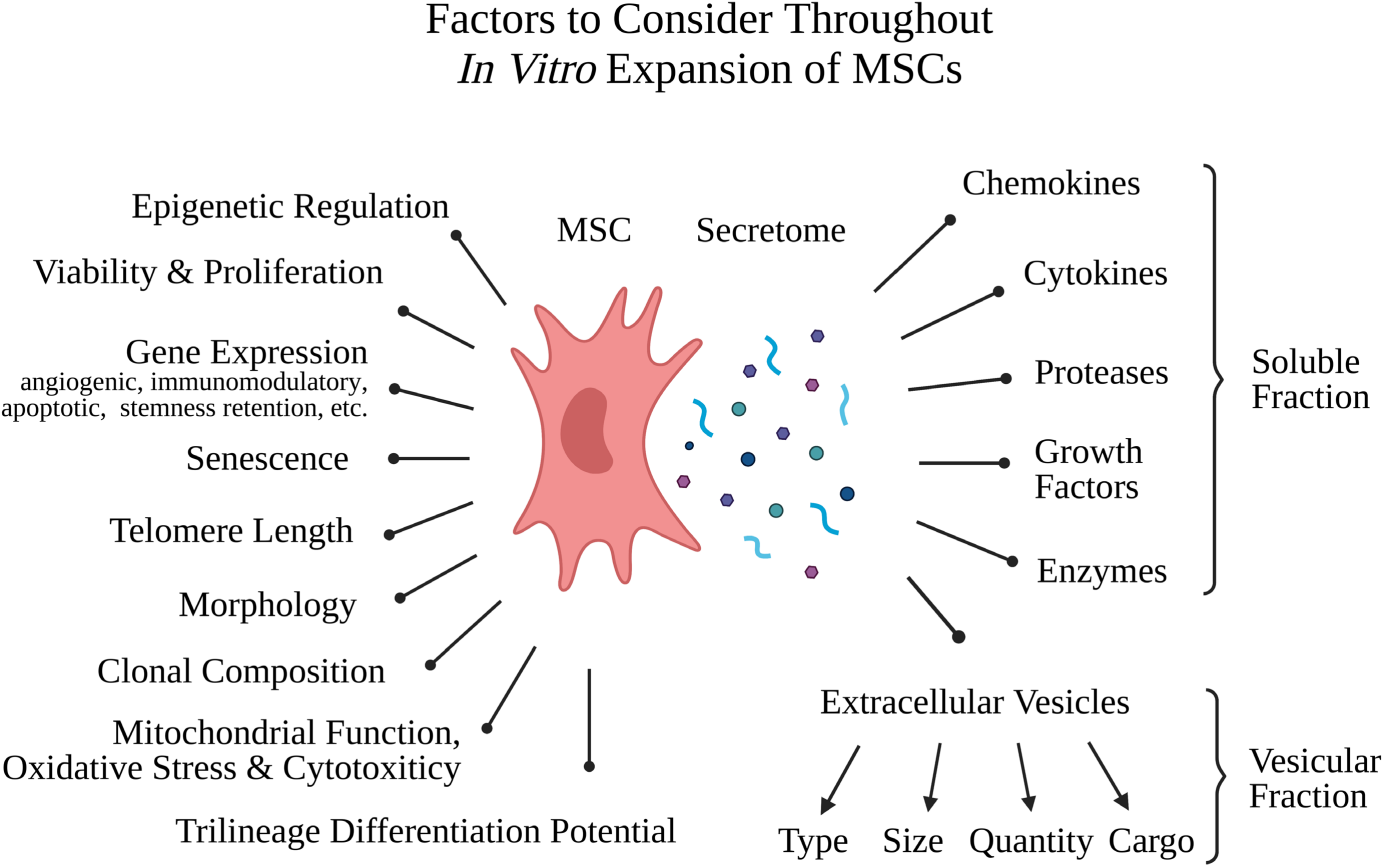
Factors to consider throughout In vitro expansion. MSCs undergo changes in epigenetic regulation, viability and proliferation, gene expression, senescence, telomere length, morphology, clonal composition, oxidative stress, mitochondrial function, cytotoxicity markers, and trilineage differentiation potential. Additionally, the secretome is also impacted by expansion, with changes occurring to the soluble fraction (chemokines, cytokines, proteases, growth factors enzymes) and the vesicular fraction (the type, size, quantity, and cargo of extracellular vesicles). Created with BioRender.com.

A series of dynamic and diverse changes occur throughout each stage of the expansion process. These changes alter the phenotypic, morphological, and functional characteristics of MSCs as well as the regenerative and immunomodulatory properties of the secretome profile. A number of factors impacted throughout the expansion process are summarized in Figure 2.

Despite variation in culture conditions, in vitro expansion alters the MSC phenotype, resulting in less diverse and therapeutically effective populations of cells. In vitro passaging has long been shown to have morphological and functional implications on differentiation potential and gene expression of BMSCs (Fehrer et al., 2006). Throughout passaging, the initial MSC population, composed of highly complex subpopulations with different functional properties, undergoes a massive clinal constriction, resulting in the selection of single clones (Selich et al., 2016). These culture‐adapted cells expanded in vitro often reflect neither the in vivo properties of the innate tissue source from which they were derived nor the functional properties exerted when reintroduced into patients (Caplan, 2019).

Like other somatic cells, MSCs can only be expanded for a limited number of cell divisions prior to entering a state of replicative arrest, one of the hallmarks of cellular senescence. Senescence is a cellular response to endogenous and exogenous stressors that can be induced by a number of stimuli, including oxidative stress, irradiation, chemicals, or replicative exhaustion. Senescence is a progressive, dynamic, and multistep process that includes chromatin remodeling, epigenetic modifications, mitochondrial alterations, and the production of a proinflammatory secretome and ends in an irreversible state of growth arrest (Neri & Borzì, 2020).

### 3.2 Onset of replicative senescence

While human diploid strains were originally estimated to replicate 40–60 times before becoming senescent (Hayflick & Moorhead, 1961), recent recommendations of achieving clinical‐grade MSCs through serial passaging suggest limiting the number of population doublings to avoid growth arrest, senescence and the possibility of spontaneous malignant transformation—albeit contested in human MSCs (Sensebé et al., 2013). While subpopulations of human MSCs (hMSCs) vary in their ability to retain regenerative properties, hMSC cultures progressively lose differentiation potential during in vitro expansion followed by a loss of proliferative potential, a key feature of senescence (Digirolamo et al., 1999). Human MSCs are estimated to undergo senescence ranging from as early as 15–40 population doublings during in vitro expansion (Banfi et al., 2000; Baxter et al., 2004; Bonab et al., 2006; Digirolamo et al., 1999; Muraglia et al., 2000). Despite several similar trends between in vitro expansion and in vivo aging, it remains unclear the extent to which in vitro expansion reflects in vivo aging or rather a by‐product of sub‐optimal in vitro culture conditions that result in a “sham” aging phenotype (Saeed & Iqtedar, 2014).

One byproduct of expansion that is an underlying mechanism in the induction of replicative senescence is the reduction of telomere length. With every cell division, telomeres shorten until they reach a critical length that triggers cell‐cycle arrest (Baird et al., 2003; Liu et al., 2020). Because telomerase enzyme activity to repair critically short telomeres is limited in somatic cells (Koliada et al., 2015), telomere length is often used as a proxy for a cell's “mitotic clock.” causing MSCs to exhibit replication‐dependent telomere shortening with age both in in vitro and in vivo (Harley, 1991; Olovnikov, 1996). In addition to replicative attrition, age‐dependent telomere shortening in vivo may also be attributed to oxidative stress, most notably high levels of reactive oxygen species (ROS) (Toussaint et al., 2000). These changes are closely tied to age‐related alterations in mitochondrial metabolism; MSCs from older donors show decreased mitochondrial membrane potential, lower mitochondrial NADH levels, and decreased absolute mitochondrial mass, collectively triggering the overproduction of ROS in MSCs (Barilani et al., 2022).

Although significant differences exist between in vitro expansion and in vivo aging, in both situations cellular modifications include decreased proliferation rates, telomere shortening, and increased proportions of senescent cells. The self‐renewing and regenerative potential of MSCs decreases with aging due to DNA damage accumulation, metabolic impairments, and mitochondrial damage (Neri, 2019; Yun, 2015). Human MSCs derived from young donors had significantly more cells at primary confluence, a greater number of population doublings until growth arrest, more days in culture prior to reaching growth arrest, more passages until growth arrest than those of old donor (Baxter et al., 2004). Furthermore, there was a strong positive correlation between absolute telomere length and the total number of population doublings occurring between primary passage and growth arrest (Baxter et al., 2004) and biological donor age and MSC population doubling time (Mareschi et al., 2006). The strong correlation between systematic reductions in telomere length and reduction in proliferation rate throughout replication support the telomere theory of cellular senescence, where chromosome shortening itself triggers an irreversible cell cycle block while maintaining other cell function.

### 3.3 Replicative senescence as a progressive and organized process

**FIGURE 3:**
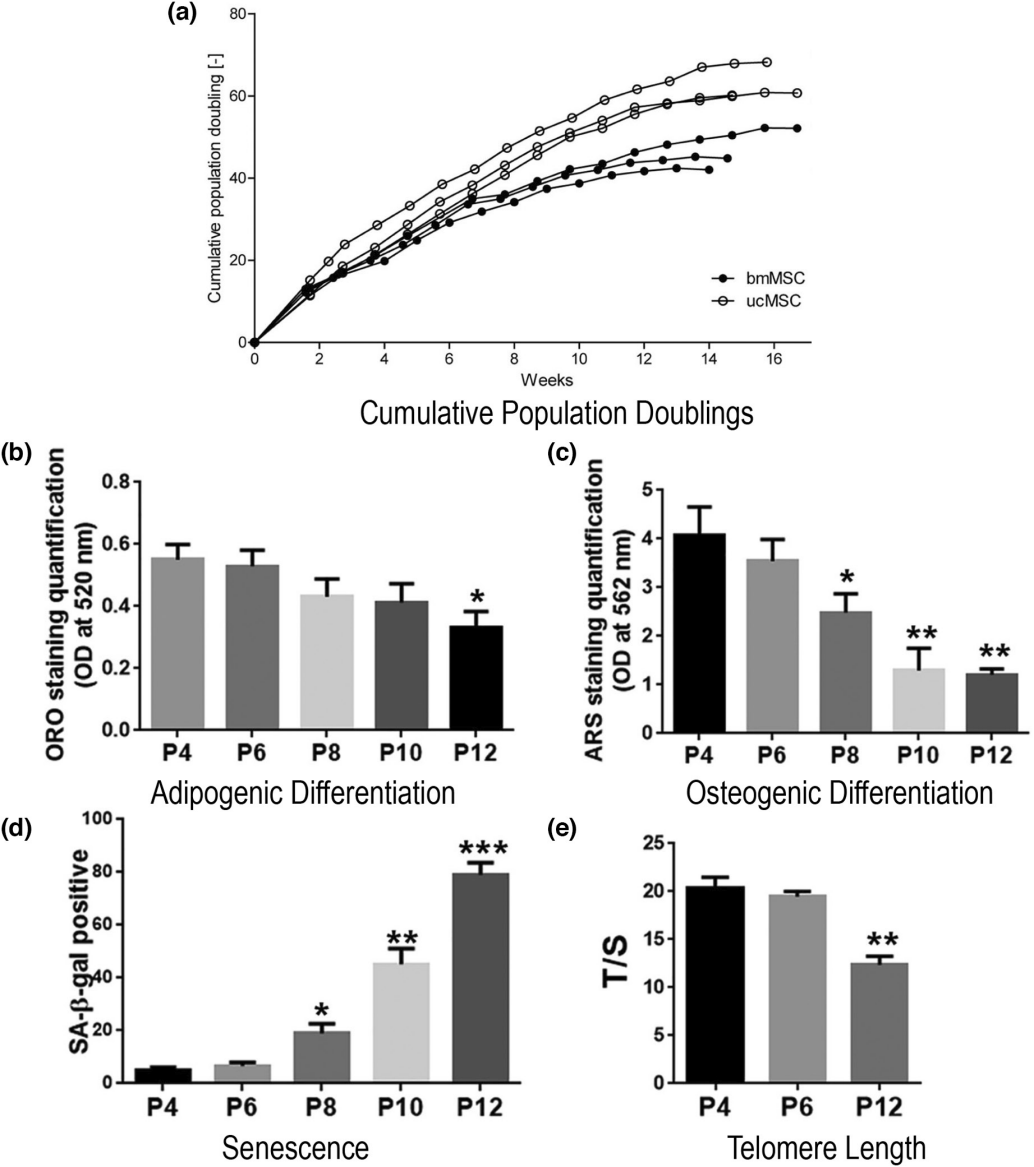
Functional changes in MSCs throughout in vitro expansion. Throughout in vitro expansion of human bone marrow and umbilical cord MSCs, there is a gradual decrease in the rate of cumulative population doublings (a), in adipogenic differentiation (b), osteogenic differentiation (c) and telomere length (e), and an increase in markers of senescence (d). (a) Growth kinetics (cumulative population doublings, CPD) of bmMSCs and ucMSCs over 17 weeks of in vitro expansion (de Witte et al., 2017). (b) Quantification of Adipogenic differentiation (ORO staining); (c) quantification of osteogenic differentiation (ARS staining); (d) quantification of senescence (percent of SA‐β‐gal staining) at P4, P6, P8, P10, and P12 (e) (Wang et al., 2021). Relative telomere length detection of MSCs in P4, P6, and P12. Data are presented as the means ± standard error of the mean, and statistically significant differences are represented as *p < 0.05, **p < 0.01, ***p < 0.001 compared with P4. *n* = 9 MSCs per passage. Figure created by combining data from de Witte et al. (2017) (a) and Wang et al., 2021 (b–e).

These changes throughout in vitro expansion appear to proceed in a sequential and organized process. (Figure 3) The altered function and morphology of MSCs is mirrored by changes in global gene expression and cell surface markers. mRNA profiling showed differential expression of 10 genes across six donor samples between early and late passages; genes involved in cell cycle, DNA replication and mitosis were significantly downregulated in senescent cells. An in‐depth analysis using MSCs from three donors revealed how these changes in mRNA expression were not restricted to senescent passages but increased during the course of replicative senescence. Additionally, upregulation of differentially expressed miRNA (hsa‐mir‐369‐5P, hsa‐mir‐29c, let‐7f) in senescent cells showed similar patterns of incremental changes over the course of in vitro expansion (Wagner et al., 2008). Additionally, the upregulation of specific miRNA has been shown to induce senescence in human cells (miR‐34a; Weilner et al., 2013).

These findings, reinforced by the progressive decline in telomere length of MSCs throughout culturing (Baxter et al., 2004) and the secretome's gradual phenotypic transition to release increasingly greater pro‐inflammatory factors (Coppé et al., 2010), point towards the onset and progression of senescence as a continuous process. As opposed to a binary conception of senescence, cells undergo a multifaceted series of changes throughout expansion with a progressive loss of regenerative capacity, corresponding to different stages of the senescence spectrum.

In addition to the complexity of phenotypic changes that MSCs undergo in long‐term culture, the time‐sensitive nature of these changes and variety of culture condition factors that influence MSCs makes developing a comprehensive understanding of changes in and functionality of MSC phenotype throughout expansion is challenging. These are further complicated by non‐uniform terminology. The plethora of different terms used to refer to the process of isolating and growing MSCs, includes “ex vivo expansion” (Gharibi & Hughes, 2012), “MSC propagation in vitro” (Drela et al., 2019), “long term culture” (Basciano et al., 2011), “serial passaging” (Lee et al., 2013) and “in vitro aging” (Geißler et al., 2012). As the terminology employed often varies by field and experimental goals (i.e., to assess replicative senescence with in vitro aging or evaluate phenotypic changes with long‐term propagation in culture), a thorough survey of the effects of MSC expansion requires an interdisciplinary approach.

## 4 PRO‐REGENERATIVE TO PRO‐INFLAMMATORY: SECRETOME SHIFT FROM THROUGHOUT IN VITRO EXPANSION SHIFTS

**FIGURE 4:**
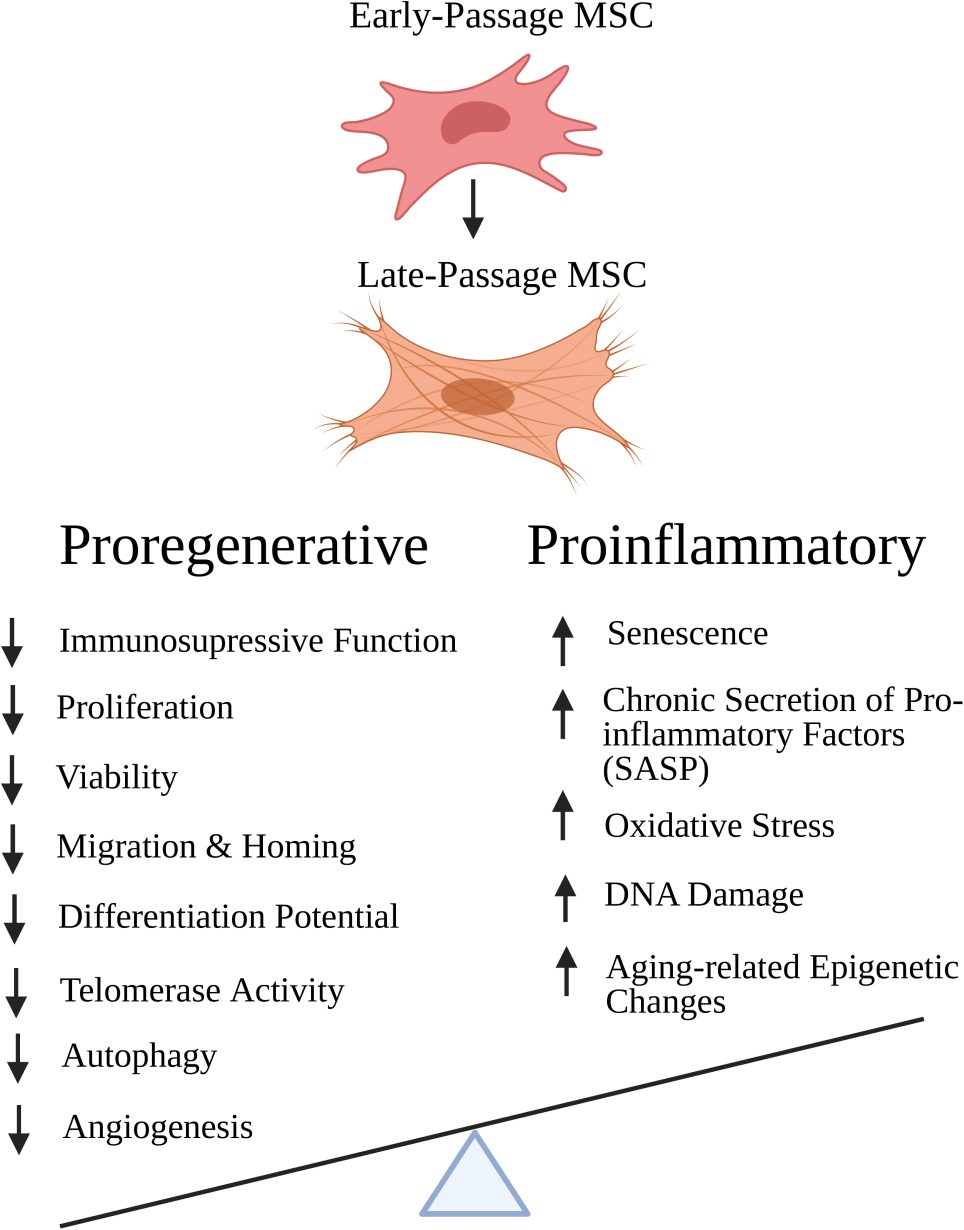
Progressive decrease in Proregenerative properties and increase in Proinflammatory factors throughout in vitro expansion of MSCs culture‐expanded MSCs exhibit a loss of functional properties including immunomod cxulatory potential, proliferation, viability, migration, homing, differentiation potential, telomerase activity, autophagy, and angiogenic potential. Changes throughout in vitro culture, hypothesized to be at the origin of these impairments, include alterations in DNA methylation profile, DNA damage accumulation, morphological abnormalities, telomere shortening, oxidative stress, senescence, and secretion of pro‐inflammatory factors. Created with BioRender.com

As MSCs age both in vivo and in vitro, their regenerative properties and immunomodulatory properties are progressively compromised (Figure 4). When comparing the efficacy of MSCs in treating acute graft‐versus‐host disease, patients with early‐passage MSCs (passages 1–2) had significantly better outcomes than those treated with later passage MSCs (passages 3–4), with increased patient survival and treatment response rates (von Bahr et al., 2012). Additionally, early passage (passage 3) MSCs showed enhanced ability to inhibit T‐cell activation relative to later passage (passages 5–7), suggesting a decrease in immunosuppressive capacity with in vitro expansion (Klinker et al., 2017). High passage MSCs exposed to human blood show an increased blood‐mediated inflammatory reaction relative to low passage MSCs, as assessed by increased platelet activation and thrombin formation in vitro (Moll et al., 2012).

Likewise, the regenerative effects of MSCs in tissue repair, which have also been attributed to their ability to home to the site of injury, are also reduced in in vitro and in vivo aged MSCs. MSCs derived from older mice show reduced CXCR4 cytokine receptor surface expression, neovascularization capacity, and migratory potential relative to younger mice. Long‐term culture expansion showed similar trends of decreased migration potential with MSCs from older mice that was accompanied by a decrease in antioxidant capacity and alteration in mitochondrial morphology and function (Geißler et al., 2012; Rombouts & Ploemacher, 2003). The complex effects of in vitro culture expansion on MSC's secretome (often studied as the senescence‐associated secretory phenotype, or SASP) have been hypothesized to abolish the regenerative process (Drela et al., 2019; Legzdina et al., 2016). This is supported by the theory that aging is characterized by a gradual decline in numerous physiological functions that ultimately resorts in organ failure and death due to both a progressive degeneration or calls and a loss of regenerative capacity (Knapowski et al., 2002).

**FIGURE 5:**
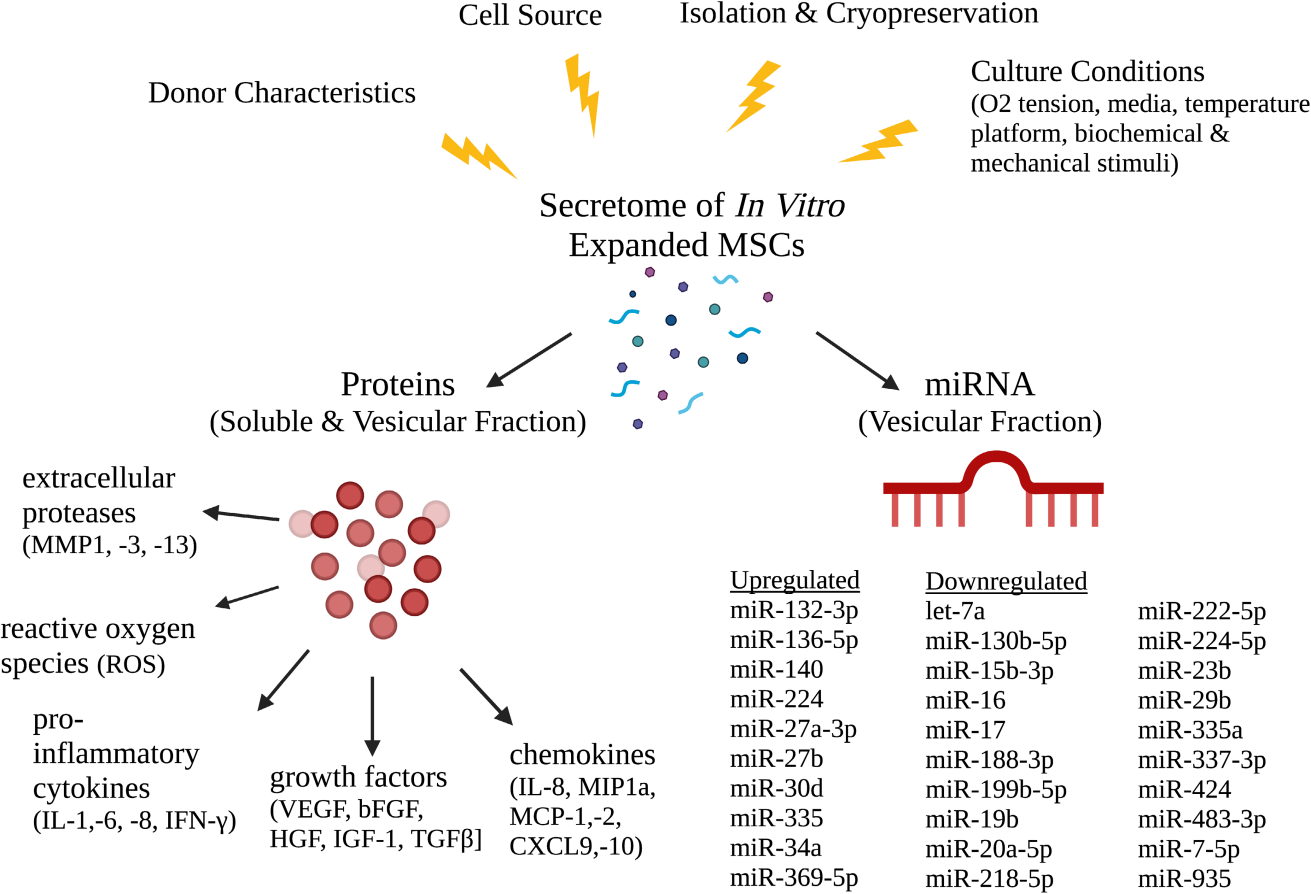
Immunomodulatory factors secreted by MSCs throughout in vitro expansion. Several immunomodulatory factors of the MSC secretome are upregulated throughout in vitro expansion, including interleukins (IL‐6, IL‐7, IL‐1a and b, IL‐13, IL‐15), chemokines (IL‐8, GRO‐a, −b, −g, MCP‐2, MCP‐4, MIP‐1a, MIP‐3a, Eoxtaxin‐3, CXCL9, CXCL10), growth factors and regulators (bFGF, HGF, KGF, VEGF, SCF, PIGF, MMP1, −3, −10), and other inflammatory factors (GM‐CSE, MIF, PGE2; Coppé et al., 2010; Zhang et al., 2021). In addition to these factors, culture‐expanded cells also secrete higher levels of reactive oxygen species (ROS), which induce oxidative stress in neighboring cells. Another component of the secretome of in vitro expanded MSCs is an alteration in the number and composition of extracellular vesicles, specifically the profile of secreted miRNA packed within exosomes. This collection of these changes in miRNA gene expression were summarized by Potter et al. (2021). Figure created using BioRender.com.

With progressive expansion and the accumulation of senescence cells, MSCs exhibit characteristics associated with chronic inflammation, marked by the secretion of pro‐inflammatory signals (Turinetto et al., 2016; Figure 5). Throughout aging, the phenotype and secretome of MSCs undergo progressive changes until the cells reach a final state of senescence. The well‐characterized, proinflammatory SASP observed in senescent cells is likely the end point of this transformation, suggesting the limitations of using a binary healthy/senescent to characterize cell states. Additionally, the number and composition of EVs secreted by MSCs is also altered throughout aging. The number of EVs secreted by MSCs increases with donor age, long‐term in vitro culture, and following senescence‐inducing stimuli (Fafián‐Labora et al., 2017; Lei et al., 2017). With aging, there is a decrease in immunologically active EVs, contributing to a progressive decrease in immunomodulatory capacity, specifically the activation of the immune system through the induction of anti‐inflammatory cytokines and T cells (Fafián‐Labora et al., 2017).

The secretion of EVs from senescent cells is hypothesized to be a major driver in the pro‐inflammatory shift that promotes the development of age‐related diseases, such as osteoporosis and vascular calcification (Prattichizzo et al., 2019; Weilner et al., 2013). EVs from young mice injected intraperitoneally into old mice significantly increased their lifespan and physical activity and reduced the expression of age‐related chronic inflammatory markers, such as IL‐1β, IL6, and TNF‐α (Wang, Yuan, & Xie, 2018). Conversely, EV cargo released from senescent cells stimulates the induction of senescence in neighboring cells via the “bystander effect” (da Silva et al., 2019). The injection of EVs derived from aged mice into healthy donors induced a mir21/mir‐217 impairment of DNMT1‐SIRT1 expression, reduced proliferation markers, and increased senescence, reinforcing the hypothesis that EVs released from senescent cells spread senescent‐inducing signals to surrounding cells via miRNA cargo (Mensà et al., 2020). Similarly, circulating EVs from the blood plasma of aged donors reduced the osteogenic potential of young MSCs (Weilner et al., 2016) and late passage MSC‐derived EVs exhibit a decreased ability to promote osteogenesis relative to early passage EVs (Lei et al., 2017). Another recent study by Fafiola et al. found adding MSC‐derived EVs from young murine models decreased levels of aging marker and players in the mTOR pathway and increased pluripotency markers in old MSC populations. Adding MSC‐derived EVs from old murine models to young MSCs produced the opposite effect (Fafián‐Labora et al., 2020).

In the cargo of these MSC‐derived EVs, a number of specific micro‐RNAs (miRNA), non‐coding RNA, and target sequences have been identified as key players in the aging progress. Pandey et al. (2011) compared the whole genome miRNA qPCR assay of young and old human donors and found 45 miRNAs were differentially expressed, constituting 5.86% of all evaluated miRNAs. Over 95% of these differentially expressed sequences were downregulated with age. Further bioinformatics analyses of the canonic pathways influenced by miRNAs showed age‐related decreases in miRNA that promote cellular movement, cell signaling, cell death, inflammatory diseases and age‐related increases associated with cellular compromise, antigen presentation, cellular growth and proliferation, cell death, and cancer. Comparisons of miRNA from MSCs from their EV derivatives showed similar trends in decreasing number of highly expressed miRNAs, suggesting that miRNAs derived from EVs likely reflect the state and characteristics of their parent cells (Lei et al., 2017).

Several studies have investigated differentially expressed MSC‐derived miRNA throughout aging. In addition to the list of differentially regulated miRNAs throughout aging as summarized by Potter et al. (2021), other miRNA have also been identified as differentially expressed throughout aging. Studies of MSC‐EVs derived from donors of different ages found increasing physiological age corresponded to downregulation of miR‐223‐5p and upregulation of miR‐127‐3p and miR‐125b‐5p with aging (Huang et al., 2019); downregulation of miR‐146a, miR‐155, and miR‐132, all of which target immunomodulator signaling pathways (Fafián‐Labora et al., 2017); and downregulation of miR‐133b‐3p and miR‐294 (Wang, Wang, et al., 2018). Other studies of in vitro expansion of MSCs identified additional differentially expressed miRNA: upregulation of miR‐1207‐5p, miRNA‐1225‐5p, miR‐150‐3p, miR1915‐3p, miR‐25‐3p, miR‐2861, miR‐3665 L, miR‐371a‐5p, miR‐4281, miR4327, miR‐762, and miR‐93‐5p (Kilpinen et al., 2016); and upregulation of hsa‐mir‐371, hsa‐mir‐369‐5P, hsa‐mir‐29c, hsa‐mir‐499, and hsa‐let‐7f (Wagner et al., 2008). In‐depth analysis of miRNA expression of one donor revealed these aging‐related effects were continuously acquired throughout in vitro culture and were not restricted to senescent passages (Lei et al., 2017).

Although MSC‐derived EVs are believed to be critical regulators of aging and immunomodulation and have shown promise in regenerative medicine and tissue engineering cell‐free therapies (for review, see Zhao et al., 2020) there has been little consistency in the identification of miRNA sequences driving age‐related changes. Just as there are a number of bioprocessing factors that influence MSC functionality and characteristics, several factors may contribute to variations in specific MSC‐derived miRNAs across studies, including type of cell aging (in vitro vs. in vivo aging), parent cell–cell contact (confluence, seeding density), MSC source (umbilical cord, bone marrow, adipose), and culture system (medium composition, cell‐adhering support, and biochemical and biophysical properties) (Maumus et al., 2020). Continuing to characterize the miRNA sequences in MSC‐EVs, targeted signaling pathways, and sources of variation in EV cargo across donors and culture conditions will further facilitate the development of miRNA‐based therapeutic interventions and design of scalable, cGMP compliant manufacturing considerations (Adlerz et al., 2020).

## 5 FUTURE DIRECTIONS

There has been growing interest in developing and expanding the use of strategies aimed at minimizing the pro‐inflammatory shift that occurs throughout in vitro expansion. The manipulation of culture conditions through the modulation of biological, biochemical, and biophysical factors, an approach often termed priming or preconditioning, can influence MSC fate, differentiation potential, and other therapeutic functions. Several priming approaches shown to improve the retention of the therapeutic efficacy, survival and function of MSCs in culture conditions include priming with (a) inflammatory cytokines or mediators (IFN‐γ, TNF‐α, IL‐1β, TGF‐ β1), (b) culturing under hypoxic conditions, (c) pharmacological drugs and chemical agents (valproic acid, all‐trans retinoic acid, dimethyloxalylglycine, etc.) (d) biomaterials and different culture conditions (3D cell culturing in scaffold/hydrogel, spheroid formation, mechanical stretch, etc.), (e) other molecules (melatonin, curcumin, lipopolysaccharide, LL‐37 cathelicidin; Alagesan et al., 2022; Hu & Li, 2018; Noronha et al., 2019). Collectively, these priming approaches have wide‐ranging effects spanning the immunomodulation, regeneration, migration, angiogenesis, survival and engraftment, anti‐apoptosis, and trilineage differentiation potential of MSCs.

Several bioprocessing techniques have also been shown to enhance the biogenesis, secretion and functional properties of MSC‐EVs. MSC‐EVs content is strongly influenced by cross‐talk between MSCs and their surrounding microenvironment, which encompasses oxygen concentration, inflammatory stimuli, stress, intracellular calcium and mechanical strain (Mullen, Williams, et al., 2022; Rani et al., 2015). Exposure to hypoxic conditions has been shown to placental MSC‐EV production by 3.3‐ and 6.7‐fold in 1% and 3% O2 conditions as well as exhibit enhanced migration and tube formation function through modification of their cargo (Salomon et al., 2013). Additionally, MSC‐EVs primed in an inflammatory microenvironment after exposure to the secretome of lipopolysaccharide or amyloid β oligomer‐activated microglia leads to the secretion of EVs with enhanced anti‐inflammatory capabilities, with miRNA profiling revealing an overexpression of target genes on the toll‐like receptor‐4 (TLR‐4) signaling pathway (Markoutsa et al., 2022). As the large‐scale production of MSC‐EVs provides significant advantages over the expansion of MSCs due to their small size, ease of production and storing, and low immunogenicity and a long circulatory half‐life (Markoutsa et al., 2022), continued research on the impact of bioprocessing culture conditions on the content of EV cargo and its behavior in vivo would benefit the development of cell‐free therapeutics.

In addition to manipulating culture conditions to maximize culture‐expanded MSCs and MSC‐EV therapeutic potential, several strategies have specifically focused on reducing MSC senescence through genetic and pharmacological interventions. Both genetic and pharmacological elimination of senescent cells in animal models have shown promise in delaying age‐related pathologies and extending the lifespan (Knapowski et al., 2002). Pharmacological approaches to reduce senescence include both senomorphic drugs (reversal of senescence) and senolytic drugs (clearance of senescent cells). The in vitro application of senolytic drugs (ABT‐263, quercetin, nicotinamide riboside, danazol, fisetin, and metformin) to human MSCs showed mixed results in decreasing molecular markers for senescence, calling for further research on dosage, potency and mechanism of action of senolytics to specifically and effectively target senescent cells within MSC populations (Grezella et al., 2018; Kim et al., 2021; Mullen, Goff, et al., 2022). Recent studies show that administration of senolytics agents (fisetin) works in a dose‐dependent manner to selectively attenuate markers of senescence such as ROS, senescence‐associated β‐galactosidase, and senescence‐associated heterochromatin foci without negatively inhibiting differentiation potential, reinforcing the potential of pharmacological elimination of senescent cells (Mullen, Goff, et al., 2022). As of November 2022, there are 16 registered clinical trials registered on https://www.clinicaltrials.gov/ (search term: “senolytic”) assessing the effectiveness of administering senolytics in conditions including osteoarthritis, Alzheimer's disease, COVID‐19, chronic kidney disease and cancer (“Home ‐ ClinicalTrials.Gov,” n.d.).

Additionally, as a critical component of aging and cellular reproductive arrest includes the progressive attrition of telomeres, upregulating telomerase activity has been harnessed as a means to combat senescence and its pro‐inflammatory secretome (SASP). The upregulation or activation of the telomerase reverse transcriptase (TERT) gene, which encodes the catalytic component of the telomerase enzyme which, is one method to activate a telomere maintenance pathway (Shay & Wright, 2019). Telomerase activity has been shown to slow telomere attrition through the de novo addition of TTAGGG repeats onto the chromosome ends. In murine models in vivo, overexpression of human TERT (hTERT) is shown to improve the fitness of epithelial barriers, delay age‐associated inflammatory and degenerative processes, preserve tissue regeneration potential and extend the median life span of. (Tomás‐Loba et al., 2008). In vitro, hTERT overexpression has been shown to extend the life span of hMSCs, attaining a population doubling level of over 80 (corresponding to 38 or greater passage numbers); hMSC‐TERT cells and hMSCs exhibit similar patterns of CD markers, multipotential differentiation capacity, morphologies and global gene expression phenotype (Twine et al., 2018; Wolbank et al., 2009). Although most studies show that hTERT cells maintain a pro‐regenerative phenotype and do not show tumorigenic potential after extensive expansion, other studies show spontaneous transformation of hASCs following 4–5 months in vitro culture and demonstrate a high rate of tumorigenicity after 3 years in culture (Wolbank et al., 2009). While the establishment of immortalized MSCs could be a helpful step in the development of cellular therapies and overcoming passaging‐dependent senescence, more research is required to better characterize the genetic differences between hMSC‐TERT and hMSCs, to verify differentiation potential and immunosuppressive effects, and to determine appropriate boundaries for in vitro culture.

## 6 CONCLUSION

The process of in vitro expansion of MSCs results in complex and diverse phenotypic changes that have a significant impact on the therapeutic potential of MSCs; both the characteristics of MSCs and their secretome profile are strongly influenced by the conditions they are exposed throughout in vitro expansion. The many ways in which MSCs are altered throughout expansion continues to gain attention in research and clinical applications. An increasing number of findings supports the hypothesis that MSCs undergo a shift from a pro‐regenerative to a chronic inflammatory phenotype and secretome throughout in vitro expansion. This pro‐inflammatory state results in a decrease in MSC therapeutic efficacy. Several specific alterations to culture conditions that more closely mimic the niche environment in vivo have been shown to retain more of the pro‐regenerative, therapeutic properties and minimize chronic inflammatory markers associated with aging. These factors include modulation of glucose and oxygen, three‐dimensional spheroid culture, co‐culturing with other cells from tissue of origin, exposure to biochemical and mechanical stimuli, pharmacological interventions, and telomerase overexpression.

While the effect of altering specific culture conditions in tightly controlled experimental conditions on MSCs have been well studied, a more comprehensive understanding of the collective changes to MSCs' phenotype and secretome profile (mRNA, miRNA, proteins secretion, survivability, proliferation, and morphology) throughout in vitro expansion would be beneficial. Specifically, a detailed assessment of these functional changes throughout culture expansion is critical to elucidating the temporal and causal relationship of the diverse factors driving the inflammatory response. Continuing to better understand the hallmarks of different stages of the spectrum of replicative senescence throughout MSC expansion, and the various ways to alter isolation and expansion conditions to maximize the therapeutic potential of the secretome of culture‐expanded MSCs will be key to the development of future MSC‐based therapies.

## AUTHOR CONTRIBUTIONS

K.M. prepared the structure of the manuscript, reviewed relevant literature, drafted all manuscript sections and generated figures. C.B. and M.S. co‐supervised the conception and design of the article, aided in the interpretation of literature, and substantively revised the manuscript. W.H. and J.H. provided consultation for the conception of the article and interpretation of literature. M.S., C.B., and J.H. provided funding to support the research.

## FUNDING INFORMATION

Research reported in this publication was supported by the AO Foundation (Miclau/Stoddart) and AO Trauma (Stoddart), and in part by the National Institute of Arthritis and Musculoskeletal and Skin Diseases (NIAMS) of the National Institutes of Health (NIH) under award number R01 AR077761 (Bahney). The content is solely the responsibility of the authors and does not necessarily represent the official views of the National Institutes of Health.

## CONFLICT OF INTEREST

Katherine Miclau has no conflicts of interest. Dr. Martin Stoddart discloses an unpaid position on the leadership for Orthopaedic Research Society (ORS), International Combined Orthopaedic Research Society (ICORS), and the Tissue Engineering and Regenerative Medicine International Society (TERMIS). Dr. Johnny Huard discloses an unpaid position on the leadership for Orthopaedic Research Society (ORS). JH discloses royalties from Cook Myosite, Inc. Dr. Chelsea Bahney discloses unpaid positions on the leadership for Orthopaedic Research Society (ORS), Tissue Engineering and Regenerative Medicine International Society (TERMIS), the Orthopaedic Trauma Association (OTA), and the AO Research and Development Commission. CB also discloses IP royalties from Iota Biosciences, Inc. for US Patent 041263 and an Associate Editor role for the Journal of Tissue Engineering and Regenerative Medicine (JTERM). These entities provided no funding for this research and there are no conflicts of interest with the work presented in this manuscript. William S. Hambright, Dr. Johnny Huard, and Dr. Chelsea Bahney are all paid employees of the non‐profit Steadman Philippon Research Institute (SPRI). SPRI exercises special care to identify any financial interests or relationships related to research conducted here. During the past calendar year, SPRI has received grant funding or in‐kind donations from Arthrex, DJO, MLB, Ossur, Siemens, Smith & Nephew, XTRE, and philanthropy. These funding sources provided no support for the work presented in this manuscript unless otherwise noted.

## Data Availability

None.
